# Host-derived gene silencing of parasite fitness genes improves resistance to soybean cyst nematodes in stable transgenic soybean

**DOI:** 10.1007/s00122-019-03379-0

**Published:** 2019-06-22

**Authors:** Bin Tian, Jiarui Li, Lila O. Vodkin, Timothy C. Todd, John J. Finer, Harold N. Trick

**Affiliations:** 1 Department of Plant Pathology, Kansas State University, 1712 Claflin Road, Manhattan, KS 66506 USA; 2 Department of Crop Sciences, University of Illinois, 1201 W. Gregory Drive, Urbana, IL 61801 USA; 3 Department of Horticulture and Crop Science, OARDC, The Ohio State University, 1680 Madison Ave, Wooster, OH 44691 USA; 4 Present Address: Innatrix Inc, 6 Davis Drive, Research Triangle Park, NC 27709 USA

## Abstract

**Key message:**

Soybean expressing small interfering RNA of SCN improved plant resistance to SCN consistently, and small RNA-seq analysis revealed a threshold of siRNA expression required for resistance ability.

**Abstract:**

Soybean cyst nematode (SCN), *Heterodera glycines*, is one of the most destructive pests limiting soybean production worldwide, with estimated losses of $1 billion dollars annually in the USA alone. RNA interference (RNAi) has become a powerful tool for silencing gene expression. We report here that the expression of hairpin RNAi constructs, derived from two SCN genes related to reproduction and fitness, HgY25 and HgPrp17, enhances resistance to SCN in stably transformed soybean plants. The analyses of T_3_ to T_5_ generations of stable transgenic soybeans by molecular strategies and next-generation sequencing confirmed the presence of specific short interfering RNAs complementary to the target SCN genes. Bioassays performed on transgenic soybean lines targeting SCN HgY25 and HgPrp17 fitness genes showed significant reductions (up to 73%) for eggs/g root in the T_3_ and T_4_ homozygous transgenic lines. Targeted mRNAs of SCN eggs collected from the transgenic soybean lines were efficiently down-regulated, as confirmed by quantitative RT-PCR. Based on the small RNA-seq data and bioassays, it is our hypothesis that a threshold of small interfering RNA molecules is required to significantly reduce SCN populations feeding on the host plants. Our results demonstrated that host-derived gene silencing of essential SCN fitness genes could be an effective strategy for enhancing resistance in crop plants.

**Electronic supplementary material:**

The online version of this article (10.1007/s00122-019-03379-0) contains supplementary material, which is available to authorized users.

## Introduction

Soybean cyst nematode (SCN), *Heterodera glycines*, is one of the most economically important biotic stresses in global soybean production, causing more than $1 billion dollars in yield losses annually in the USA alone (Koenning and Wrather 2010). Although the use of resistant cultivars has been the most effective management strategies for SCN controls in the past, sources of genetic resistance used in commercial soybean production are limited and various virulent nematode populations are beginning to emerge as resistance breaks down (Klink and Matthews 2009). Breeding for resistance to SCN remains highly challenging due to the limited availability of SCN-resistant genes and because the mechanism of soybean resistance to SCN is still unclear (Gillet et al. 2017; Liu et al. 2017). Alternative novel approaches are needed to provide resistance to this widespread and destructive soybean pest.

Since it was first demonstrated in *Caenorhabditis elegans* (Fire et al. 1998), RNA interference (RNAi) has emerged as an efficient tool for knocking down transcript levels of specific target genes. Over the past two decades, many studies have successfully applied the strategy of host-delivered RNAi to reduce pathogen infection. The effectiveness of host-derived RNAi strategies has been demonstrated in controlling a wide range of plant parasitic insects, fungi, and nematodes (Bolognesi et al. 2012; Cheng et al. 2015; Fairbairn et al. 2007; Govindarajulu et al. 2015; Huang et al. 2006; Sanju et al. 2015; Yadav et al. 2006; Zhang et al. 2015). Furthermore, RNAi experiments targeting SCN have shown similar encouraging results. For SCN infection, pre-parasitic second-stage juveniles (pre-J2) of SCN initiate the infection by invading the plant vascular cylinder. Syncytia are induced on soybean roots by parasitic (par)-J2 and J3 SCN that usually have a swollen body shape and rounded tail compared to pre-J2. J4/adult females with a near lemon shape keep feeding on plant roots until eggs develop (Sobezak and Golinowski 2009). For initial efforts to use RNAi to control SCN, soaking *H. glycines* infective juveniles in RNAi solutions of a conserved ribosomal gene (*Hg-rps-23*) resulted in sterile and sick nematodes as shown by positive Sytox fluorescence (Alkharouf et al. 2007). Transgenic soybean lines expressing dsRNA of a gene coding for Major Sperm Protein of *H. glycines* reduced eggs g^−1^ root tissue up to 68% (Steeves et al. 2006). *In planta* expression of SCN homologs of several genes with lethal mutant phenotypes in *C. elegans* significantly reduced the ability of *H. glycines* females to mature (Klink et al. 2009). Previous studies from our laboratory reported diminished *H. glycines* egg production by up to 95% using soybean hairy roots expressing siRNAs for several additional genes involved in reproduction and/or development (Li et al. 2010, 2009). It appears that a potentially large reservoir of essential and specific nematode genes may be available for use in host-derived RNAi strategy against nematode infection.

Limitations of the host-derived RNAi strategy include efficient delivery of siRNA molecules to target pathogens and the effectiveness of these molecules in triggering the RNAi pathway in those pathogens (Li et al. 2011). To date, the effectiveness of nematode control using this strategy has been variable (Klink et al. 2009; Kumar et al. 2017; Sindhu et al. 2009; Vieira et al. 2015). To achieve new and stable resources for SCN resistance in soybean breeding programs and to demonstrate consistent effects against SCN, we obtained stable transgenic soybean plants expressing RNAi silencing vectors and analyzed the small interfering RNA(siRNA) expression levels in multiple lines via small RNA-seq. In the present study, two targeted SCN developmental genes were evaluated, *H. glycines* gene HgY25 (NM_062040), encoding a beta subunit of a coatomer (COPI) complex, and HgPrp-17 (AF113915), encoding a pre-mRNA splicing factor. Studies on the homologous genes in *C. elegans* indicated that both genes are required for fertility, adult viability, proliferation and meiotic development (Kamath et al. 2003; Nickel et al. 2002; Kerins et al. 2010). In addition, both genes were identified as suitable targets for RNAi-based resistance in our previous study with composite plants (Li et al. 2010). Analysis of the siRNA sequence profile via deep sequencing confirmed that the dsRNAs of both HgY25 and HgPrp-17 were expressed in transgenic plants and were cleaved into overlapping 20–24 nt siRNA molecules. The two most effective RNAi transgenic lines of each target gene were selected from approximately 16 transgenic events, and homozygous T_4_ generations were obtained. Bioassays confirmed that each of these stable transgenic lines continued to display significant reductions in numbers of SCN cysts and eggs. Additionally, and to the best of our knowledge, we report for the first time a correlation between host-induced siRNA expression and the level of SCN resistance. Our data suggest a threshold level of siRNA molecules was required to significantly reduce SCN populations feeding on the host plants. Our results add to the growing body of evidence supporting the use of host-induced RNAi as an effective strategy for SCN management and breeding programs. In addition, the siRNA expression data presented here provide valuable information for advancing plant pest or pathogen control approaches more generally through the expression of target siRNA sequences of pests or pathogen genes in host plants.

## Materials and methods

### Cloning of HgY25 and RNAi construct

**Fig. 1:**
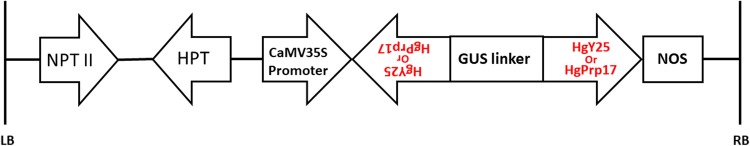
The structure of the RNAi constructs used for transformation. The backbone vector is pBI121. The red indicates the fragment positions of either HgY25 or HgPrp17 introduced into the construct as inverted repeats. The NPT II and HPT cassettes are selection markers for kanamycin resistance and hygromycin resistance, respectively (color figure online)

*HgY25* (Genbank Accession No. CB824330) from *H. glycines* was identified from datasets in nematode.net. Using *H. glycines* HG type 2.7 cDNA as template, a 292-bp fragment of *HgY25* was obtained by PCR with specific primers Y25-F and Y25-R (Table S1). Y25-F and Y25-R were also used to amplify the *HgY25* gene from HG type 7 and HG type 1.3.5.6.7 of *H. glycines*. The 3′ race of *HgY25* was performed according to the instructions of GeneRacer® Kit (Invitrogen, Carlsbad, CA). We recovered a 1101 bp of *HgY25* (Genbank Accession No. HM369132), including the 872 bp coding region and 229-bp 3′ UTR. A 383 bp fragment of the *HgPrp17* gene was obtained with primers Prp17-F and Prp17-R (Table S1). The binary vector pANDA35HK was a kind gift from Ko Shimamoto (Miki and Shimamoto 2004). The pANDA35HK RNAi vector has an RNA interference cassette under the control of the constitutive 35S cauliflower mosaic virus (CaMV35S) promoter. This vector contains the *nptII* gene that confers resistance to kanamycin, and a hygromycin phosphotransferase gene (*hpt*) both driven by a CaMV35S promoter. Cloning of the PCR products into pANDA35HK was performed as described previously (Li et al. 2009). Briefly, both PCR products were first ligated into pGEM-T Easy vector and then subcloned into pENTR4 vector by *Eco*RI restriction sites. Subsequently, the pENTR4 vector carrying *HgY25* was recombined with pANDA35HK vector using LR clonase (Invitrogen, Carlsbad, CA). The resulting RNAi expression vectors, pANDA35HK_Y25 and pANDA35HK-Prp17 contained the 292 bp and 383 bp gene fragments, respectively, in complementary orientation, separated by 930-bp GUS linker fragment, and driven by the CaMV35S promoter (Fig. 1).

### Stable transformation of soybean mediated by bombardment

The soybean cultivar “JackX” (Jack X PI417138) was used for transformation (kind gift from Dr. Wayne Parrott at the University of Georgia). This line had been chosen as the model genotype for SCN gene-silencing experimentation, is susceptible to SCN, and is amenable to transformation. Soybean somatic embryos were initiated and maintained according to Finer and McMullen (1991). Soybean embryos of cultivar “JackX” were bombarded with DNA-coated tungsten particles with a Particle Inflow Gun (Finer et al. 1992). Following the transformation procedure, hygromycin was used in the embryo proliferation medium to select for tissue expressing the plasmid pANDA35HK Y25 and Prp17 containing the *hpt* gene. Selection and regeneration for soybean transformation were performed as described by Trick et al. (1997).

### Identification of transgenic soybean plants by PCR, reverse transcription PCR (RT-PCR), and qRT-PCR

Plant DNA from young soybean leaves was extracted by E.Z.N.A® Plant DNA Kit (Omega Bio-tek Inc., Norcross, GA, USA) following the manufacturer’s instruction. The genomic DNA (gDNA) quality was examined by the NanoDrop ND-1000 Spectrophotometer (Nanodrop Technologies, Wilmington, DE, USA) and PCR with gene-specific primers for the Ribosomal S21 gene (Rib-F and Rib-R) (Li et al. 2010). The Gus-F1 and Gus-R2 primers situated within the *GUS* linker of the RNAi construct, and they were used to identify the presence of sense and antisense fragments in expression RNAi constructs. The Gus-R2 primer was paired with Y25-R or Prp17-R for amplification of GmY25 or GmPrp17 sense fragments, and Gus-F1 was paired with Y25-R or Prp17-R for amplification of GmY25 or GmPrp17 antisense fragment. PCR cycling comprised an initial step at 94 °C for 3 min, followed by 32 cycles at 94 °C for 30 s, 58 °C for 30 s, and 72 °C for 45 s.

Total RNAs from transgenic soybean plants were isolated using TRIzol reagent (Invitrogen, CA, USA) according to manufacturer’s instructions. The eggs of nematodes feeding on plants were pooled and collected with three biological replications in each experiment, and total RNA was extracted following the protocol described by Tian et al. (2016). For reverse transcription PCR (RT-PCR), 1 μg of total RNA was treated with DNase I (Promega, Madison, WI) and reverse-transcribed using Reverse Transcription System kit (Promega, Madison, WI) following the manufacturer’s instructions. The detection of target gene expression was conducted using the same protocol as described above. Quantitative PCR was performed on the CFX96 Touch™ Real-Time PCR Detection system (Bio-Rad, Hercules, CA, USA) using iQ™ SYBR® Green Supermixes (Bio-Rad). The qPCR program was set up with one cycle for template denaturation and hot start Taq activation at 95 °C for 2 min, then 40 cycles of 95 °C for 5 s, and 60 °C for 20 s extension step with dissociation. The conserved *H. glycines* β-actin (AF318603) was used as the internal control in nematodes (Tian et al. 2016). The RT-qPCR reaction was done following the manufacturer’s protocol with three biological and three technical replicates for each sample. The relative target gene expression was calculated using the 2^–∆∆CT^ method (Livak and Schmittgen 2001) for all SCN samples. All primers used are listed in Table S1.

### Small RNAs sequencing from transgenic plants

All transgenic plants were grown in the greenhouse at 26 °C and 16/8 day/night photoperiod. During the bioassays, either leaves and/or roots were collected, flash-frozen in liquid nitrogen, and stored at − 80 °C for further analysis. Plant tissues were then freeze-dried and total RNA was isolated as previously described (Tuteja et al. 2009). A small RNA library was prepared with an Illumina Small RNA version 1.5 Sample Prep Kit and sequenced by synthesis using the Illumina GAIIx at the Keck Center of the University of Illinois. After trimming the 3′ adapter, sequences retaining greater than 16 nt were compared to obtain the unique sequences and the number of occurrences of each distinct sequence. Alignments of small RNAs to target genes sequences were performed using Bowtie v1 (Langmead et al. 2009) allowing no mismatches. Bowtie outputs were further filtered to retain sequences from 18 to 25 nt to plot size distributions, alignments to the target sequence, and calculate read per million (RPM) values (reads aligned per million total sequence reads). All plant samples selected in bioassays for small RNAs sequencing are listed in Tables S2–S4.

### SCN bioassay

To explore suppression of nematode reproduction in transgenic plants expressing dsRNA of HgY25 and HgPrp17 genes, transgenic seeds were planted individually in D40 Deepots (Stuewe and Sons, Inc., Corvallis, OR) containing a SCN HG type 7-infested sand-soil mix with approximately 4500 eggs per 100 cm^3^ of soil. The nematode population originated from a naturally infested commercial soybean field in Cherokee Co., KS, and was maintained on a susceptible soybean variety, KS3406RR, under greenhouse conditions. The non-transgenic, susceptible soybean cultivar JackX was used as negative control. Bioassays were performed on transgenic plants and negative controls as described by Tian et al. (2016). In initial T_1_ generation screening bioassays, a total of 12 seeds (four biological replicates in each of three bioassay experiments) for each event were randomized and planted in individual D40 Deepot containing the SCN infested soil. The same number seeds of susceptible cultivar JackX and resistant cultivar KS4313N were also included in each assay as controls. In subsequent SCN bioassays, 15 (T_2_–T_3_ generation) to 20 (T_3_–T_4_ generation) plants of transgenic lines whose expression were confirmed by both PCR and RT-PCR, and which showed significant effects in T_1_ generation bioassays, were evaluated in one (T_2_–T_3_ generation) to three (T_3_–T_4_ generation) independent experiments. After five weeks post-inoculation, the soil was washed from the plant roots, and the cysts and eggs were collected and counted as previously described (Li et al. 2009). The root dry weights were measured for each root after one week in a 50 °C drying room. Numbers of cysts and eggs were counted under the microscope and normalized as cysts/g root, eggs/g root. Data from bioassays were subjected to analysis of variance with the GLM procedure in SAS (SAS Institute, Cary, NC, USA).

## Results

### *Hereodera glycines* HgY25 and HgPrp17 genes and RNAi vector construction

A 1101-bp nucleotide sequence of the *HgY25* gene (GenBank accession No. HM369132) was cloned and obtained from nematode cDNA by RACE-PCR. This sequence included the 876-bp open reading frame (ORF) region encoding 291 amino acids and a 225-bp 3′ un-translated region (UTR). The selected 292-bp sequence, used in the RNAi construct, was highly conserved among different *H. glycines* populations (Fig. S1a) and displayed more than 99% homology among three populations of HG type 2.7, HG type 7, and HG type 1.3.5.6.7. The *HgY25* gene had limited similarity with beta subunit of the COPI complex from *C. elegans* and other species (Fig. S1b). The *H. glycinesPrp-17* gene (GenBank accession No. AF113915) encodes a pre-mRNA splicing factor, and it was selected based on previous results in transgenic hairy roots (Li et al. 2010). To efficiently generate specific siRNAs against both SCN genes in transgenic soybean plant, the inverted repeats of 292 bp HgY25 and 383 bp HgPrp17 were independently cloned into the pANDA35HK vector (Miki and Shimamoto 2004) by Gateway cloning. Therefore, the resulting RNAi expression vectors pANDA35HK-Y25 and pANDA35HK-Prp17 contained both antisense and sense fragments of the target genes, separated by a 930-bp GUS linker fragment, and regulated by the constitutive CaMV35S promoter and the NOS terminator (Fig. 1).

### Molecular analysis of transgenic soybean plants expressing RNAi constructs

**Fig. 2:**
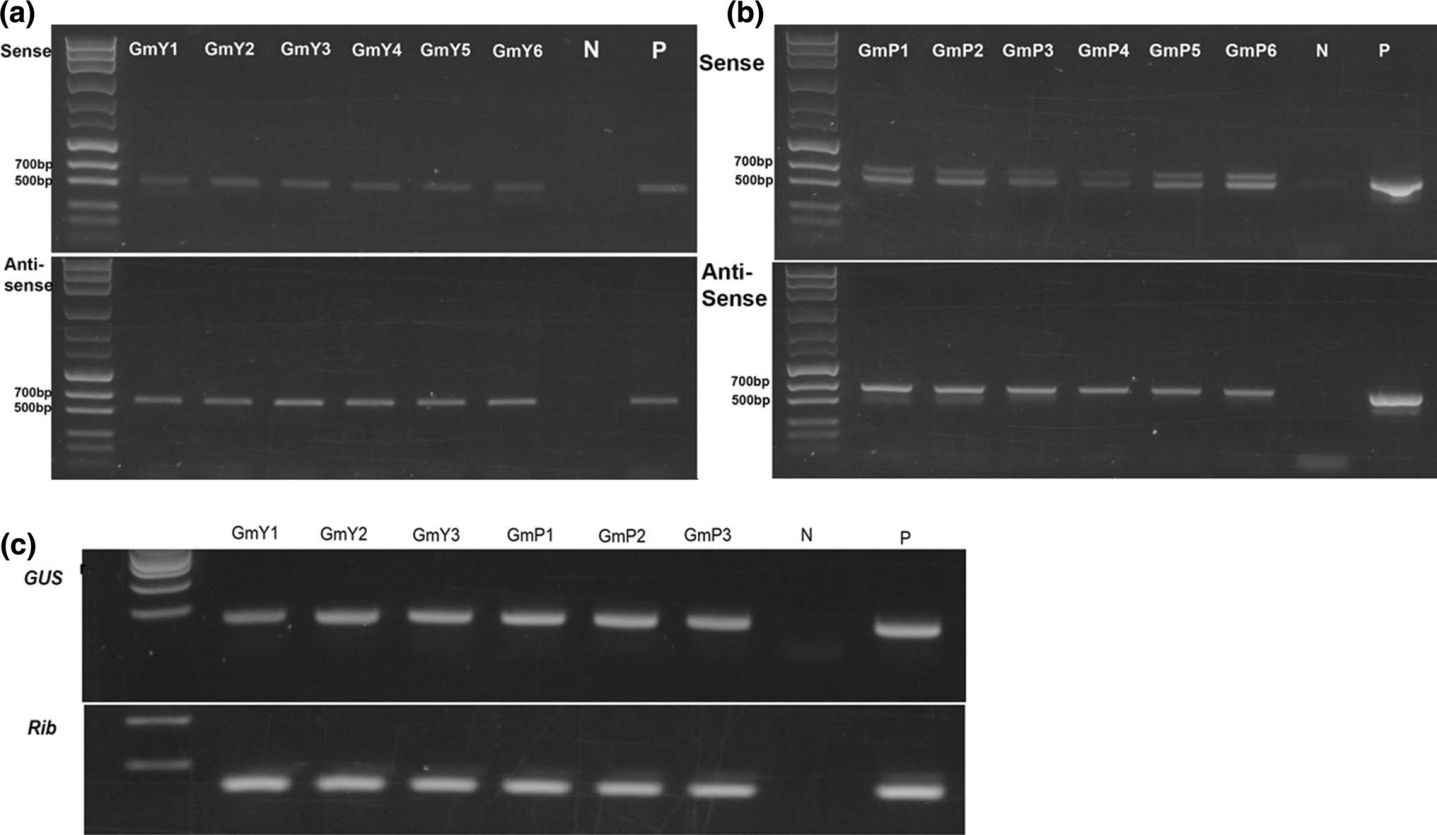
Molecular analysis of RNAi-resistant transgenic (GmY25 and GmPrp17) soybean plants. **a** The amplification results of pANDA35HK-Y25 vector in soybean genomic DNA. The GUS-F1 and Y25 reverse primer pair and GUS-R2 and Y25 reverse primer pair were used to confirm both sense and antisense fragments presenting in the transgenic lines. GmY1–6 were six independent transgenic lines; N was non-transgenic soybean sample; and P was a control plasmid, pANDA35HK-Y25. **b** The amplification results of pANDA35HK-Prp17 vector in soybean genomic DNA. The GUS-F1 and Prp17 reverse primer pair and GUS-R2 and Prp17 reverse primer pair were used to confirm both sense and antisense fragments presenting in the transgenic lines. GmP1–6 were six independent transgenic lines; N was non-transgenic soybean sample; and P was a control plasmid, pANDA35HK-Prp17. **c** RT-PCR analysis to detect the expression of GUS linker on RNAi construct; three lines from pANDA35HK-Y25 (GmY1–3) and pANDA35HK-Prp17 (GmP1–3) were exhibited; N was blank control, P was a control plasmid in GUS and was cDNA from non-transgenic soybean in Rib, which is soybean conserved *Rib* gene (Ribosomal-S21, CF921751) as control

Soybean embryos were transformed with RNAi constructs containing either pANDA35HK-Y25 or pANDA35HK-Prp17 by particle bombardment to generate transgenic soybean plants as previously described (Steeves et al. 2006). All T_0_ plants were tested for the presence of the gene of interest (GOI) and the hygromycin resistance gene by PCR. T_1_ seeds were harvested from a total of 9 RNAi GmY25 and 7 RNAi GmPrp17 transgenic events (Table 1). Furthermore, genomic PCR analysis indicated that both sense and antisense fragments were detected in samples of T_1_ transgenic plants (Fig. 2a, b), and RT-PCR for the *GUS* linker demonstrated that both RNAi constructs were expressed at the transcriptional level (Fig. 2c).

**Table 1:** Initial screening of SCN bioassays for all transgenic events (T_1_ seeds) for the elite line selection

Transgenic event	Average reduction in eggs/root g (mean ± SEM)
GmY25E5	50% ± 10.6%^a^
GmY25E7	44% ± 3.7%^a^
GmY25E12	59% ± 6.4%^a^
GmY25E13	58% ± 4.0%^a^
GmY25E1	6% ± 2.2%
GmY25E3	13% ± 2.7%
GmY25E4	26% ± 14.4%
GmY25E6	0 ± 3.6%
GmY25E10	11% ± 11.3%
GmPrp17P1	72% ± 9.2%^a^
GmPrp17P9	47% ± 6.2%^a^
GmPrp17P6	53% ± 8.1%^a^
GmPrp17P8	69% ± 2.2%^a^
GmPrp17P7	24% ± 6.2%
GmPrp17P2	− 2% ± 3.4%
GmPrp17P19	8% ± 4.7%

Bioassay counts include only positive transgenic plants identified by PCR analysis. The reduction percentage is presented as mean ± standard error of mean (SEM) based on three independent bioassay experiments and four biological replications for each transgenic line in each experiment, with experiments treated as a random effect

^a^Indicates that *t* test was significant at *P* ≤ 0.05

### Improved SCN resistance in soybeans transformed with RNAi constructs

**Fig. 3:**
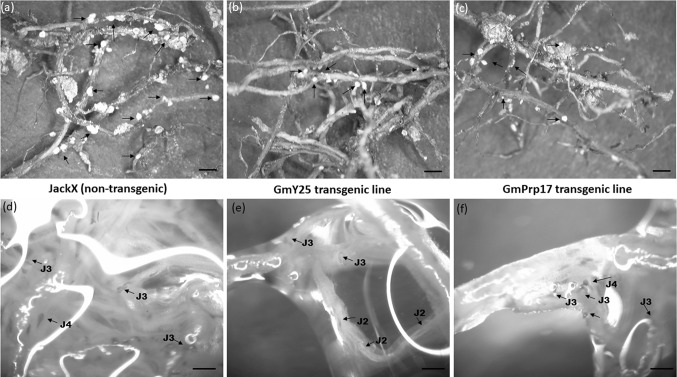
Comparison of SCN population feeding on transgenic soybean and controls five weeks after inoculation. The homozygous transgenic plants from GmY25 (T_4_) and GmPrp17 (T_5_) were used. **a**, **d** Were non-transgenic controls; **b** and **e** were transgenic GmY25 RNAi plant with less cysts and juveniles on roots; **c** and **f** were transgenic GmPrp17 RNAi plants with less cyst and juveniles on root. **a**–**c** Cysts were observed on the soybean roots under dissection microscopy; arrows indicated cysts. **d**–**f** Fuchsin staining of soybean roots for SCN visualization; arrows indicated all stages of juveniles. The scale bar represents 1 mm

The phenotypes of the transgenic soybeans transformed with either of the two constructs resembled those of the non-transgenic cultivar JackX, which was the cultivar used for transformation. Some plants of the GmY25 lines, which displayed reduced vigor and/or were smaller than non-transgenic plants in the T_1_ generation, were subsequently withdrawn from further testing. To identify whether SCN reproduction was affected by the soybean transformation process, bioassays using soil infected with *H. glycines* HG type 7 and incorporating GOI-free transgenic (non-transformed segregated soybean identified by PCR) and non-transgenic JackX seeds as negative controls were performed in the greenhouse. Prior to the bioassay, the presence and expression of pANDA35HK-Y25 and pANDA35HK-Prp17 constructs were confirmed by PCR and RT-PCR for each individual transgenic plant. An initial screening of all T_1_ transgenic lines was conducted to select elite transgenic plants for further analysis. The initial screening bioassay results indicated that several lines showed significant differences in cysts/g root and eggs/g root (cyst and egg density, respectively) between transgenic plants and negative controls (data not shown). Four events of GmY25 and four events of GmPrp17 showed significant reduction (*P* < 0.05) for SCN egg numbers, compared to the negative control. At least five transgenic plants showed more than a 50% reduction in eggs/g root (Table 1). Visibly fewer cysts were observed on the homozygous GmY25 and GmPrp17 transgenic roots, compared to negative controls (Fig. 3).

**Fig. 4:**
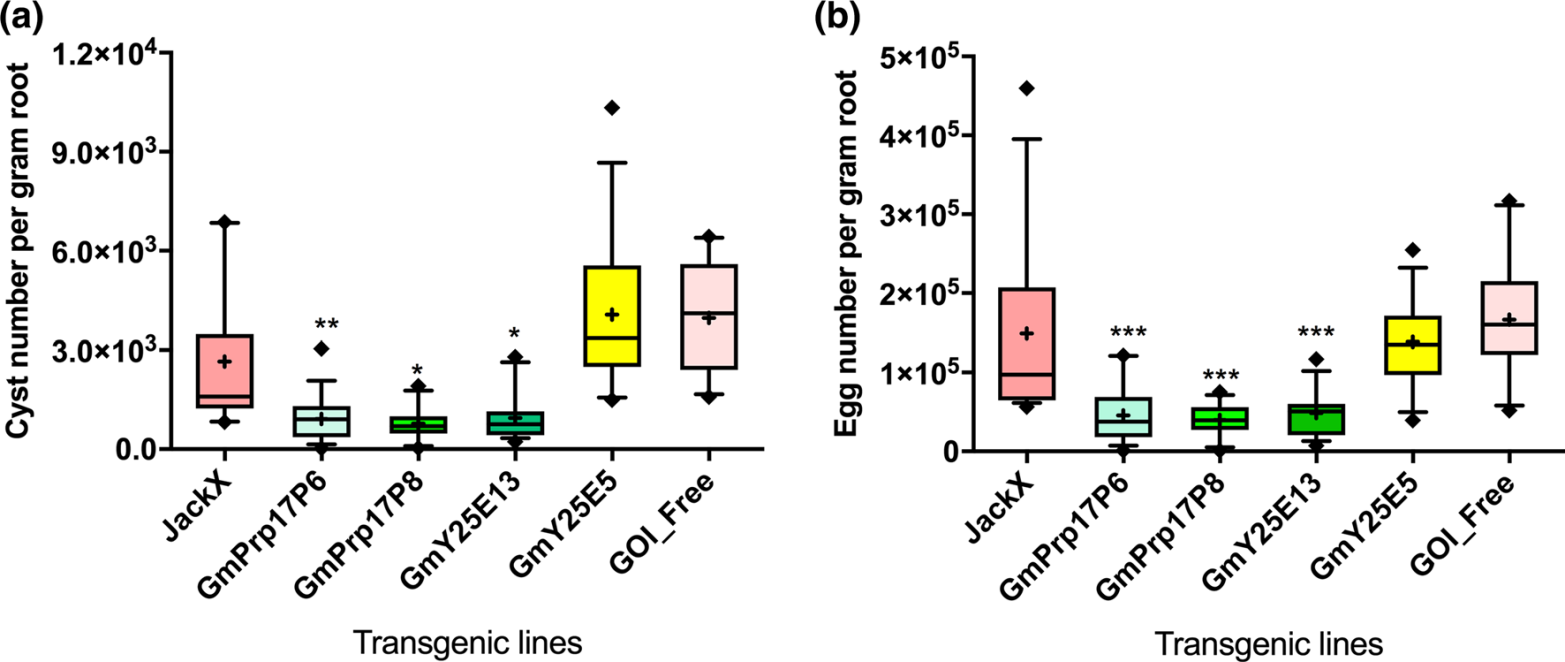
Comparison of SCN cyst (**a**) and egg (**b**) densities developing on transgenic soybean roots and controls (*n* = 15). The T_2_ and T_3_ generation transgenic lines were used for GmY25 and GmPrp17, respectively. JackX was non-transgenic soybean, and the GOI-free line was negative for target gene by PCR detection. The mean value was shown as “ + ”. The asterisks are significantly different from control JackX at *p* < 0.05 (*), *p* < 0.01(**), and *p* < 0.001(***), respectively

Based on the above results, two events of each RNAi construct (namely, GmY25E13, GmY25E5, GmPrp17P6, and GmPrp17P8) were selected for further analyses to investigate SCN resistance. The four GmY25 and GmPrp17 transgenic lines were taken to the T_2_ and T_3_ generation, respectively. To identify whether the elite transgenic soybean plants affected *H. glycines* reproduction, SCN bioassays were performed by growing these transgenic soybean plants, along with non-transgenic JackX plants as negative controls, in SCN HG type 7-infested soil. Both the GmPrp17P6 and P8 events showed a significant reduction in SCN populations averaging 69.3% and 73.3% in the egg density, respectively, compared to the control (Fig. 4, Table S5). Of the two GmY25 transgenic events evaluated (Fig. 4, Table S5), the GmY25E13 transgenic line showed a significantly reduced SCN population by 67.4% in the egg density. However, the GmY25E5 event did not affect the SCN population compared to the controls. The GOI-free plants, which were negative for target gene by PCR detection, showed similar SCN population levels as non-transgenic plants.

**Fig. 5:**
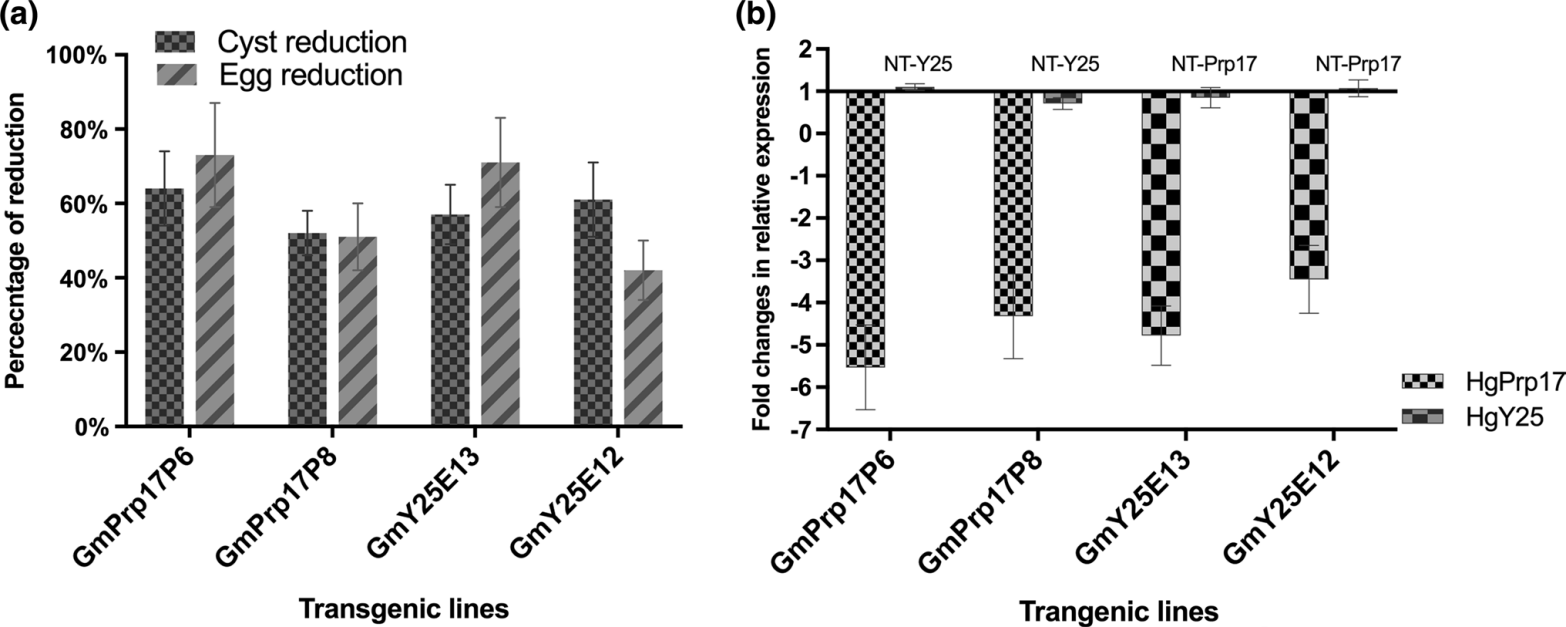
Four homozygous lines (GmY25 and GmPrp17 are T_3_ and T_4_ generation) demonstrated reductions on SCN population with down-regulated targeted genes. **a** Two homozygous lines for each GmY25 (*n* = 60) and GmPrp17 (*n* = 60) showed significantly consistent reduction of SCN population compared to non-transgenic controls in three independent bioassays. Error bars represent the standard error of the mean based on three independent bioassays, with experiment treated as a random effect. **b** qRT-PCR was used for quantitating the expression of target genes in SCN eggs collected from soybean roots, and the expression level was normalized to *beta-actin* gene in *H. glycines*. It showed the specific down-regulation of target HgY25 and HgPrp17 at transcription levels. The relative expression level of the non-target (NT) gene in each experiment remained unchanged. Error bars represent the standard error mean between three independent bioassay replicates

Two homozygous transgenic lines (GmY25E12 and GmY25E13) of GmY25 were advanced to the T_3_ generation, and two homozygous lines (GmPrp17P6, and GmPrp17P8) of GmPrp17 were taken to the T_4_ generation by self-pollination. To confirm homozygous lines with a single transgene insertion obtained, PCR analyses showed that the transgenes of all progenies in two generations were stably inherited. The transgene insert was confirmed by the segregation analysis using T_1_ generation (data not shown). To demonstrate consistent resistance to SCN, bioassays in three independent experiments for each homozygous transgenic line were performed for these generations also showed significant reductions in cyst and egg densities consistently ranging from 42 to 73% (Fig. 5a, Table S6).

### Down-regulation of HgY25 and HgPrp17 transcripts in nematodes feeding on transgenic soybeans with corresponding RNAi constructs

To determine whether the reduced SCN populations on the advanced transgenic lines (Fig. 5a) was due to the RNAi effects of inducing suppression of the targeted genes, total RNAs extracted from nematode eggs which were developed on homozygous transgenic and non-transgenic soybean roots at five weeks post-inoculation were analyzed for transcript abundance in each sample by qRT-PCR. Both targeted genes, HgY25 and HgPrp17, had down-regulated mRNA transcripts in nematode eggs collected from transgenic plant roots with the corresponding RNAi constructs (Fig. 5b). The highest (5.5 fold) reduction was observed on SCN feeding on GmPrp17P6 plants, whereas SCN on other events showed at least a 3.4-fold reduction of transcripts with targeted genes. The decrease in the transcript levels of both parasite fitness genes was statistically significant (*P* < 0.05), whereas the transcript levels of non-target genes were not statistically changed (Fig. 5b). As expected, all the target genes displayed specific mRNA down-regulation in SCN populations recovered from transgenic plants with corresponding RNAi constructs.

### Detection of specific siRNAs in transgenic soybeans

To evaluate the expression of siRNAs in transgenic plants, small RNA-seq libraries were prepared from leaf tissues and were sequenced by Illumina technology. The distribution of total unique siRNA sequences showed the highest abundance around the 21-nt length (Fig. S2), which was the same pattern as the dominant size of endogenous siRNA sequences of soybean reported in previous studies (Sun et al. 2016; Tian et al. 2017; Tuteja et al. 2009; Wang et al. 2013).

**Fig. 6:**
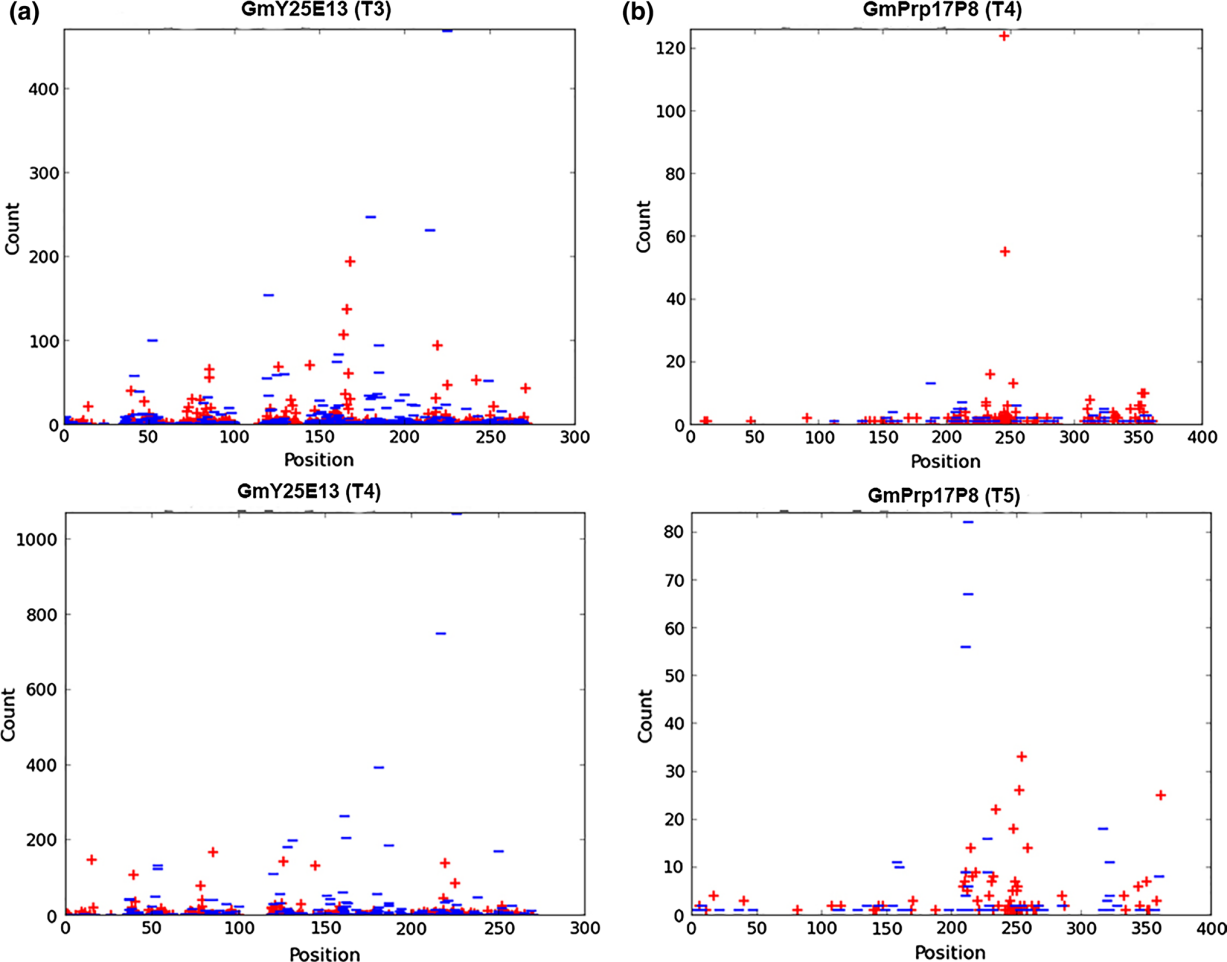
The expressed siRNA distribution on target fragments of RNAi constructs in the transgenic plants GmY25E13 (**a**), and GmaPrp17P8 (**b**). The similar patterns were also observed from different generations of the same RNAi constructs. Counts and positions of siRNAs with lengths between 18–25 nucleotides that had a 100% match with either the positive (+ ) or negative (−) strand of the Y25 or Prp17 targets are shown

Two RNAi constructs produced different siRNA profiles (Fig. 6). For the HgY25 gene (Fig. 6a), most siRNAs were produced from 125 to 270 nt region of the HgY25 fragment with the highest counts in the 150–200 nt region. There were two further hot spot regions from 30–60 nt and 70–100 nt for HgY25 siRNA. The HgPrp17 gene (Fig. 6b) produced more concentrated regions at 200–260 nt and 310–350 nt. The data also indicated that siRNAs in HgPrp17 had more unified patterns than the HgY25. Compared across generations and diverse individual plants, the patterns for siRNAs expressed by RNAi constructs appeared to be conserved through generations, although the abundance of each siRNA varied in different samples (Fig. 6).

**Fig. 7:**
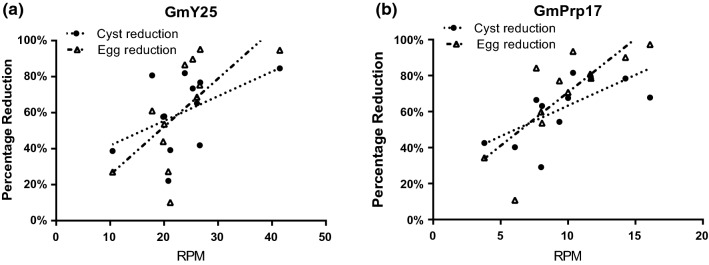
The putative relationships between siRNA expression (RPM) of RNAi constructs and the effectiveness of SCN resistance (percentage reduction in cyst and egg numbers) for 12 homozygous transgenic plants each for events GmY25E13 (**a**) and GmPrp17P6 (**b**). Linear regression equations for GmY25E13 were Y = 28.12 + 1.37X (*P* = 0.1079, *r*^2^ = 0.24) for cysts and Y =  − 1.09 + 2.67X (*P* = 0.0162, *r*^2^ = 0.45) for eggs. Linear regression equations for GmPrp17P6 were Y = 29.21 + 3.40X (*P* = 0.0174, *r*^2^ = 0.45) for cysts and Y = 11.57 + 5.91X (*P* = 0.0025, *r*^2^ = 0.61) for eggs

To investigate the potential relationship between the siRNA expression and SCN resistance, RNAs from individual heterozygous transgenic plants that showed different performance from the SCN bioassays as shown in Fig. 4 were used to prepare small RNA libraries. As shown in Table 2 for nine plants, the SCN reduction was higher in plants with higher levels of small RNAs that matched the target (target RPM). Based on their performance in the bioassays (Fig. 5a), an independent experiment using RNAs from homozygous transgenic lines GmY25E13 and GmPrp17P6 was conducted. A total of 12 libraries from each transgenic soybean line were constructed and analyzed (Table S4). As shown in Fig. 7, it can be inferred from the data set that moderate to high levels of resistance to SCN [i.e., a reduction > 70% in SCN cyst and egg densities (Niblack et al. 2009)] were achieved with increasing small RNA levels, implying that a threshold level of target siRNAs needs to be expressed.

**Table 2:** Small RNAs sequencing for target gene expression on individual transgenic plants (T_2_ generation for GmY25E13 plants, and T_3_ generation for GmPrp17P6 and P8 plants were investigated) and corresponding effects on SCN

Transgenic lines	Individual plant #	RPM both strand	Cyst reduction percentage	Egg reduction percentage
GmPrp17P6	1	845	65	69
	2	152	60	74
	3	46	29	35
GmPrp17P8	1	191	53	68
	2	51	5	36
GmY25E13	1	204	50	78
	2	210	65	66
	3	739	83	82
	4	701	78	78

## Discussion

Soybean production in the Midwestern USA is severely constrained by SCN, and the threat continues to spread (Mitchum 2016). The utilization of RNAi to engineer resistance to SCN provides alternative sources and strategies for advanced breeding. In vitro RNAi has been successfully used to silence target genes of plant parasitic nematodes (Dutta et al. 2014; Urwin et al. 2002), including *H. glycines* (Bakhetia et al. 2008). The objective of a host-derived RNAi strategy is to suppress nematode genes by delivery of the dsRNA and/or siRNA through the feeding apparatus, so that the development and/or reproduction of nematodes is disrupted and completion of their life cycle inside the plant host is prevented. For host-derived RNAi approaches, siRNAs for gene suppression are delivered continuously because obligate parasitic nematodes have to feed on their host plants throughout their life cycle (Li et al. 2011). This strategy was first demonstrated in transgenic tobacco where 90% suppression of root knot nematodes was obtained by targeting two housekeeping genes (Yadav et al. 2006). Likewise, silencing of the major sperm protein in transgenic soybean successfully impaired the life cycle of cyst nematodes (Steeves et al. 2006). Since then, this host-derived RNAi strategy has been illustrated to reduce populations of various parasitic nematodes with different hosts (Dinh et al. 2014; Dutta et al. 2015; Klink et al. 2009; Kumar et al. 2017; Li et al. 2010; Lourenço-Tessutti et al. 2015; Papolu et al. 2013; Xue et al. 2013). The degree of reduction in nematode populations feeding on host plants is highly variable, however, depending on target genes, and host plant and parasite species. Minimal impacts on nematode populations have also been reported (Fairbairn et al. 2007; Kyndt et al. 2013; Patel et al. 2010; Sindhu et al. 2009; Vieira et al. 2015).

In this study, stable transgenic soybean lines targeting the SCN parasite fitness genes HgY25 and HgPrp17 were developed. Homozygous lines of each resulted in a better than 70% decrease in nematode egg densities. Based on high-throughput sequencing of small RNAs, a correlation between host-derived expression and resistance performance indicated that a threshold for siRNA expression needed to be achieved for effective suppression of parasite nematodes.

Nematode housekeeping genes are frequently targeted for RNAi suppression; however, targeting genes conserved in all eukaryotes may result in secondary effects that induce abnormal phenomena in the plant host (Bakhetia et al. 2008; Li et al. 2011; Schüssler et al. 2008). In the present study, no deviation from the normal growth phenotype was observed in the GmPrp17 transgenic lines. However, in some of GmY25 lines, smaller plants were observed. The germinated transgenic plants had shorter hypocotyls and fewer lateral roots than the non-transgenic plants; furthermore, the transgenic plants had fewer or almost no root hairs. This morphology may have resulted from potential off-target effects on the soybean host plant. Therefore, only GmY25 plants with normal phenotype were selected for advanced experimentation in this study.

Small RNA sequencing of transgenic plants expressing RNAi vectors for nematode resistance could provide insight into populations of siRNA species generated, and their utility for silencing SCN genes effectively. The siRNA sequencing results also indicated that a soybean dicer-like protein (DCL) was cleaving the *H. glycines* genes’ dsRNAs into 19–24 nt nucleotides, with similar patterns as observed for endogenous siRNA. Our results showed that the predominant size of siRNAs was 21-nt, the same as the dominant size of endogenous siRNA sequences of soybean reported by Tuteja et al. (2009), demonstrating that the cleaving mechanism of soybean DCL was likely the same for endogenous and exogenous genes. The target genes in nematodes feeding on transgenic soybean plants were correspondingly suppressed to a significant extent (Fig. 5b), thus providing evidence of effective delivery of siRNA and/or dsRNA into nematodes. The distribution of target siRNA with the same constructs followed similar patterns over generations. Based on smRNA sequencing of individual transgenic plants, it was hypothesized that siRNA levels were related to resistance. The RNA-seq analysis of siRNA *in planta* strongly suggested a threshold of RNAi construct expression level was related to the SCN resistance of the transgenic plant (Fig. 7). Either of the two RNAi constructs needed to reach the threshold concentration in transgenic plants to bring about effective reductions (defined as > 70% in present study) in nematode numbers. The results of this study support a growing body of evidence indicating that a host-derived RNAi strategy can be an effective approach for SCN management. The quantity of siRNA produced in the host plant is one of the major factors limiting the success of resistance. Future studies will investigate ways to increase siRNA expression, using high expression promoters, and/or vectors in soybean, or by manipulating siRNA amplification pathways. In addition, stacking two or more RNAi cassettes may provide host plants with additional, as well as more durable, levels of resistance against parasitic nematodes.

### Author contribution statement

BT and HNT designed the experiments and wrote the manuscript. BT designed and conducted experiments including SCN bioassays with statistical analyses, real-time PCR with data analyses, and prepared the RNA-seq samples. JL cloned the HgY25 gene and constructed vectors. LOV performed the RNA-seq and assisted the data analyses. TCT supported the SCN bioassay and participated in statistical analyses and revised the manuscript. JJF developed the T_0_ stable transgenic soybean lines and revised the manuscript. HNT designed the experiments, coordinated project, analyzed data, and wrote the manuscript.

## Electronic supplementary material

Below is the link to the electronic supplementary material.

Supplementary file1 (DOCX 2463 kb)

Supplementary file2 (DOCX 13 kb)

Supplementary file3 (XLSX 18 kb)

Supplementary file4 (XLSX 17 kb)
