# Supplementary material for: Host-derived gene silencing of parasite fitness genes improves resistance to soybean cyst nematodes in stable transgenic soybean
Source: Theor Appl Genet. 2019 Jun 22;132(9):2651–62. doi: 10.1007/s00122-019-03379-0 (PMC6707959; doi:10.1007/s00122-019-03379-0)
Supplement: Supplementary file 1 — Supplementary file1 (DOCX 2463 kb) [file 122_2019_3379_MOESM1_ESM.docx]

Figure S1 Sequence analysis of the cloned *HgY25* gene (a) sequence alignment of RNAi construct sequence with the *HgY25* gene in multiple SCN populations; (b) phylogenetic analysis of the COPI complex gene in different species.
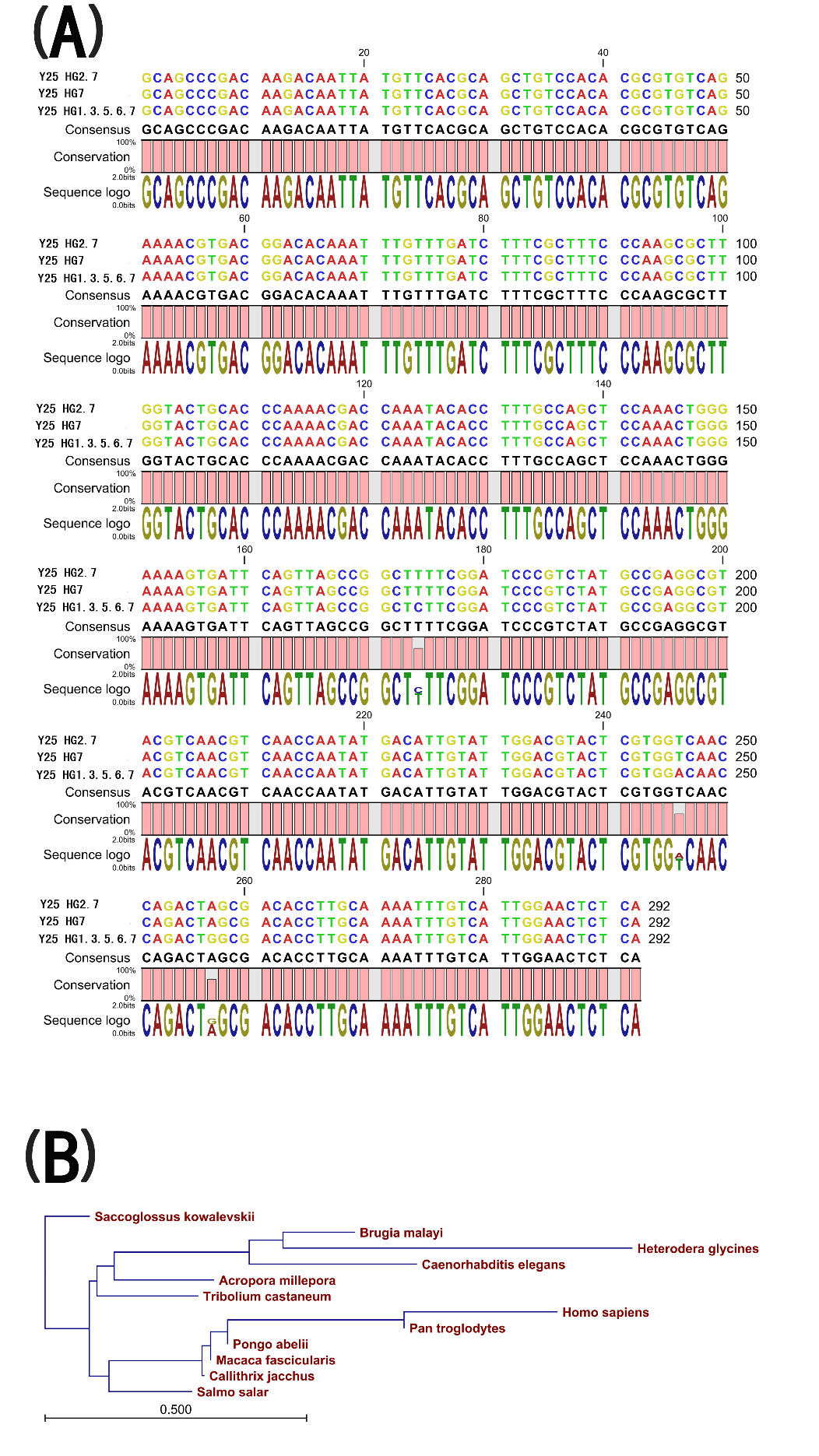


Figure S2 Length distribution of siRNA produced by GmY25 and GmPrp17 transgenic plants. The number of unique siRNA sequences is shown as well as the total count for each siRNA sequence.

**
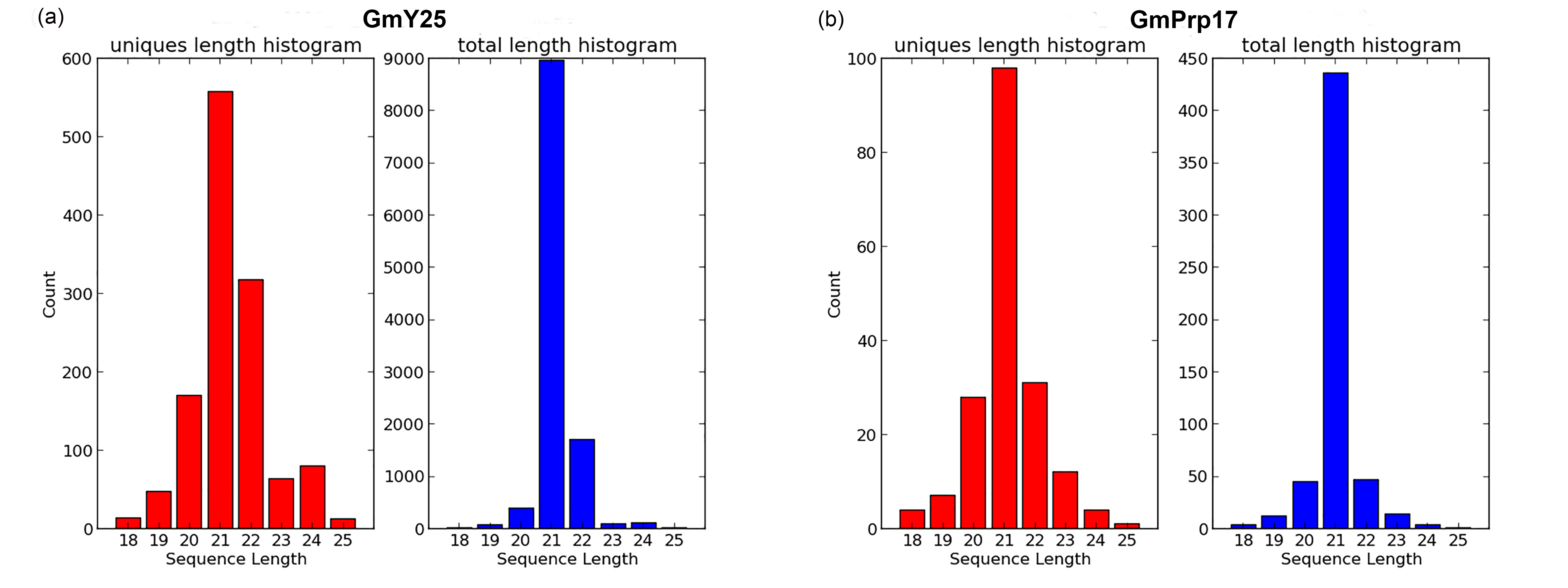
**
