# Supplementary material for: Host-derived gene silencing of parasite fitness genes improves resistance to soybean cyst nematodes in stable transgenic soybean
Source: Theor Appl Genet. 2019 Jun 22;132(9):2651–62. doi: 10.1007/s00122-019-03379-0 (PMC6707959; doi:10.1007/s00122-019-03379-0)
Supplement: Supplementary file 2 — Supplementary file2 (DOCX 13 kb) [file 122_2019_3379_MOESM2_ESM.docx]

Table S1 All primer sequences used in this study

| Primer name | Sequences (5’ -3’) | Function |
| --- | --- | --- |
| Y25-F | GCAGCCCGACAAGACAAT | Amplify *H. glycines* Y25 gene |
| Y25-R | TGAGAGTTCCAATGACAAAT |  |
| Gus-F1 | CACGTAAGTCCGCATCTTCA | Paired with reverse primers of each gene for RNAi vector confirmation |
| Gus-R2 | GTATCAGTGTGCATGGCTGG |  |
| Prp17-F | CAATCGAATTGTCCTTTTCCA | Amplify *H. glycines* HgPrp17 gene |
| Prp17-R | CATTTTGATTACATTGTCCCATC |  |
| Rib-F | CTAAGATGCAGAACGAGGAAGG | Amplify soybean ribosomal S21 gene (CF921851) to check genomic DNA quality |
| Rib-R | GAGAGCAAAAGTGGAGAAATGG |  |
| HgY25-realF | CGATCACTGAGGACGACTTGG | Realtime PCR to detect *H. glycines* HgY25 gene |
| HgY25-realR | AACGAAGCACGGCACTCTC |  |
| HgPrp17-realF | CCTACGATACGCTTGGCTATG | Realtime PCR to detect *H. glycines* HgPrp17 gene |
| HgPrp17-realR | ATTTATCACTCTCTTTCGCTTCTG |  |
| HgACT-realF | CGGCATGGGACAGAAGGA | Realtime PCR to detect *H. glycines* β-actin gene |
| HgACT-realR | CGTCAGAATACCACGCTTGGA |  |
| lnkF | GCGTGGTGATGTGGAGTATTGC | Amplify the *GUS* linker for detection |
| lnkR | TCGCTGATGGTATCGGTGTGAG |  |
